# SMAR1 inhibits Wnt/β-catenin signaling and prevents colorectal cancer progression

**DOI:** 10.18632/oncotarget.25093

**Published:** 2018-04-20

**Authors:** Nandaraj Taye, Aftab Alam, Suvankar Ghorai, Deya Ghosh Chatterji, Apoorva Parulekar, Devraj Mogare, Snahlata Singh, Pallabi Sengupta, Subhrangsu Chatterjee, Manoj Kumar Bhat, Manas Kumar Santra, Prabhakar Budha Salunkhe, Susan Kling Finston, Samit Chattopadhyay

**Affiliations:** ^1^ National Centre for Cell Science, Pune 411 007, India; ^2^ SRM University, Tamil Nadu, Kattankulathur 603 203, India; ^3^ NKP Salve Institute of Medical Sciences, Nagpur, 440 019, India; ^4^ Indian Institute of Chemical Biology (CSIR), West Bengal, Kolkata 700 032, India; ^5^ Department of Biophysics, Bose Institute, Kolkata 700 054, India; ^6^ Amrita Therapeutics Limited, Ahmedabad 380 054, India

**Keywords:** β-catenin, conditioned medium, peptide, SMAR1, Wnt3a

## Abstract

Reduced expression of Scaffold/Matrix Attachment Region Binding Protein 1 (SMAR1) is associated with various cancers resulting in poor prognosis of the diseases. However, the precise underlying mechanism elucidating the loss of SMAR1 requires ongoing study. Here, we show that SMAR1 is highly downregulated during aberrant Wnt3a signaling due to proteasomal degradation and predicted poor prognosis of colorectal cancer. However, substitution mutation (Arginine and Lysine to Alanine) in the D-box elements of SMAR1 viz. “RCHL” and “RQRL” completely abrogated its proteasomal degradation despite Wnt3a activity. SMAR1 inhibited Wnt/β-catenin signaling by recruiting Histone deacetylase-5 to β-catenin promoter resulting in reduced cell migration and invasion. Consequently, reduced tumor sizes in *in-vivo* NOD-SCID mice were observed that strongly associated with suppression of β-catenin. However, loss of SMAR1 led to enriched H3K9 Acetylation in the β-catenin promoter that further increased Wnt/β-catenin signaling activities and enhanced colorectal cancer progression drastically. Using docking and isothermal titration calorimetric studies we show that small microbial peptides viz. AT-01C and AT-01D derived from *Mycobacterium tuberculosis* mask the D-box elements of SMAR1. These peptides stabilized SMAR1 expression that further inhibited metastatic SW480 colorectal cancer cell migration and invasion. Drastically reduced subcutaneous tumors were observed in *in-vivo* NOD-SCID mice upon administration of these peptides (25 mg/kg body weight) intraperitoneally. Taken together our structural studies, *in-vitro* and *in-vivo* results strongly suggest that the D-box elements of SMAR1 represent novel druggable targets, where the microbial peptides hold promise as novel colorectal cancer therapeutics.

## INTRODUCTION

Colorectal cancer (CRC) is among the most deadly forms of cancer with increasing incidence in patients less than 50 years of age without any family history [1]. Development and growth of CRC depends on genetic mutations, epigenetic alterations, active oncogenes, loss of tumor suppressor genes and defects in signaling pathways [2–4]. Most of the CRCs arise as a polyp due to aberrant crypt formation that results into early adenoma and progressing through advanced adenomas [5]. Although prevention or early detection is possible through regular screening, its advanced metastatic stages are difficult to treat. Despite the promise of immune-oncology and targeted therapies, most CRC tumors are treated with standard therapeutic interventions including surgery, radiotherapy, and/or chemotherapy [6]. Thus, identification of novel genes involved or deregulated in CRCs may provide important insights for development of more sustainable therapeutic interventions. Here, we identified SMAR1 as a novel tumor suppressor gene in CRC.

In this study, we discuss the important inhibitory role of SMAR1 with respect to Wnt/β-catenin signaling. SMAR1 is a tumor suppressor gene that incorporates less CD44 splice variants, increases NFκB activities and inhibits TGF-β pathway thereby suppressing further tumorigenesis [7, 8]. SMAR1 also retards tumor growth by positively regulating p53 activation and arrest the cell at G2/M phase of the cell cycle [9]. In addition, SMAR1 also promotes p53 to downregulate VEGF and restrains endothelial cell migrations required for angiogenesis [10]. SMAR1 overexpression also prevent epithelial to mesenchymal transition and attenuates migration of breast cancer cells [11]. In contrast, loss of SMAR1 has been observed in higher grades of breast cancers causing malignancies [12]. Our earlier research has demonstrated the effectiveness of a 33-mer peptide of SMAR1 to significantly reduce cellular proliferation and tumor growth both *in-vitro* and *in-vivo* [13]. Anti-cancer agents like prostaglandins increase SMAR1 transcription that further suppress cell proliferation and migration [14].

Therefore, the ability to stabilize SMAR1 may prove crucial to improve therapeutic outcomes for CRC patients. We have undertaken an investigation relating to SMAR1 stability and its tumor suppressor function in CRC. Because β-catenin is active in more than 70% of CRCs, a potential therapeutic approach to inhibit β-catenin activities either molecularly or pharmacologically appears promising [15, 16]. Research has shown that various small molecule compounds [17, 18] and microbial peptides show great promises as anti-cancer therapeutics by elevating tumor suppressor functions [19, 20]. Here, we also unveil novel microbial peptides with potential to increase SMAR1 activities in the inhibition of Wnt/β-catenin signaling in CRC. In this context, we have studied the loss of SMAR1, its biological tumor suppressor function and clinical implications in CRC associated with Wnt/β-catenin signaling.

## RESULTS

### SMAR1 is downregulated in Wnt signaling driven CRC

**Figure 1:**
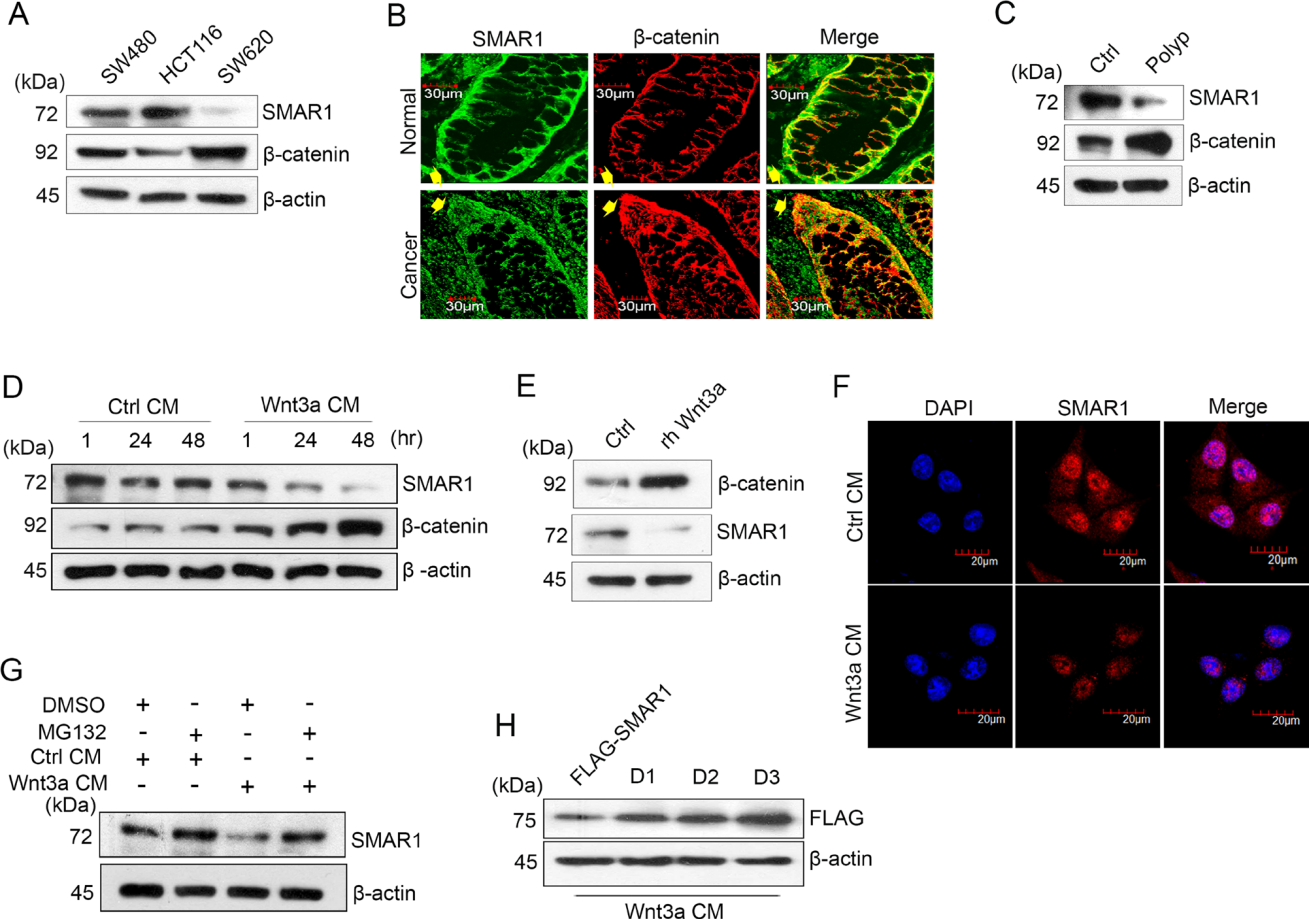
SMAR1 is downregulated in Wnt signaling driven CRC (**A**) Expression of SMAR1 and β-catenin in various CRC cell lines. (**B**) Confocal staining of colon tissue sections co-stained with SMAR1 and β-catenin antibodies. Arrow shows the basal portion of the colon crypt. The scale bar used in the confocal experiment represents 30 μm. (**C**) Expression of SMAR1 and β-catenin levels in mouse colon tissues (polyp vs normal adjacent tissue). (**D** and **E**) Expression of SMAR1 and β-catenin upon stimulating HCT116 cells with Wnt3a CM or rh Wnt3a ligand (200 ng/mL). (**F**) Confocal staining of SMAR1 after Wnt3a CM stimulation in HCT116 cells. The scale bar used in the confocal experiment represents 20 μm. (**G**) SMAR1 expressions after treating HCT116 cells with both Wnt3a CM and 10 μM MG132 drug. (**H**) SMAR1 expression in FLAG-SMAR1, D1, D2 and D3 expressing cells after Wnt3a CM stimulation.

Aberrant Wnt signaling is one of the most frequent causes of increased β-catenin expression in CRCs [8]. In this study, we observed significant downregulation of SMAR1 in a panel of β-catenin expressing CRC cells, patient-derived colon tissues at the base of the crypt and mouse colon polyps (Figure 1A–1C and Supplementary Figure 1A and 1B). Conversely cells and tissues with stable expression of SMAR1 correlated with lower expression of β-catenin, as revealed from the Western blot and immuno-fluorescence studies. These findings suggest an inverse correlation between SMAR1 and β-catenin in CRCs. To further elucidate the role of Wnt signaling in SMAR1 regulation, we stimulated HCT116 cells with Wnt3a Conditioned Medium (CM) or recombinant human (rh) Wnt3a ligand. Stimulation with Wnt3a resulted in a significant downregulation of SMAR1 with a concomitant rise in the β-catenin levels (Figure 1D and 1E). Downregulation of SMAR1 is also confirmed from the confocal studies during Wnt3a stimulation (Figure 1F). However, constitutive activation of β-catenin with RFP-β-catenin overexpression (kind gift from Jomon Joseph, NCCS) or treatment with a Glycogen Synthase Kinase 3-Beta (GSK3-β) inhibitor, LiCl [21] failed to downregulate SMAR1 (Supplementary Figure 1C and 1D). Wnt3a CM stimulation also failed to suppress SMAR1 mRNA levels, which prompted us to examine the proteasomal degradation of SMAR1 upon Wnt3a stimulation (Supplementary Figure 1E).

SMAR1 protein stability was observed upon treatment with a proteasomal inhibitor, MG132 10μM with or without Wnt3a CM stimulation (Figure 1G). Earlier we reported the presence of two D-box elements viz, “RQRL” and “RCHL” in SMAR1 [22] (Supplementary Figure 1F) that are conserved in various species (Supplementary Figure 1G). A protein with a D-box element has an RXXL sequence (X can be any amino acid) that interacts with a ubiquitin ligase and results in proteasomal degradation [23]. We reasoned that if Wnt3a stimulation contributes to SMAR1 proteolysis in CRC, the mutations in its D-box elements could bypass proteasomal degradation. Therefore, we used single (D1 as “RQRL” + “ACHA” and D2 as “AQRA” + “RCHL”) and double (D3 as “AQRA” + “ACHA”) mutant constructs of SMAR1 by substituting the arginine and leucine residues by alanine (Supplementary Figure 1H). We then assessed SMAR1 proteasomal degradation upon Wnt3a activation. Interestingly, we found that the double mutants are resistant to proteasomal degradation upon Wnt3a CM stimulation, while the single mutants let to partial SMAR1 proteolysis (Figure 1H). Further observations suggest that cells transfected with wild type FLAG-SMAR1, where the D-box elements are left in native form showed the greatest degree of SMAR1 proteasomal degradation. Further research is needed to identify if any ubiquitin ligases are involved in the proteasomal degradation of SMAR1 through the D-box elements during Wnt3a stimulation. These results support a novel association between SMAR1 and Wnt/β-catenin signaling, in which Wnt3a activation triggers the proteasomal degradation of SMAR1 in CRC.

### SMAR1 inhibits Wnt/β-catenin signaling pathway

**Figure 2:**
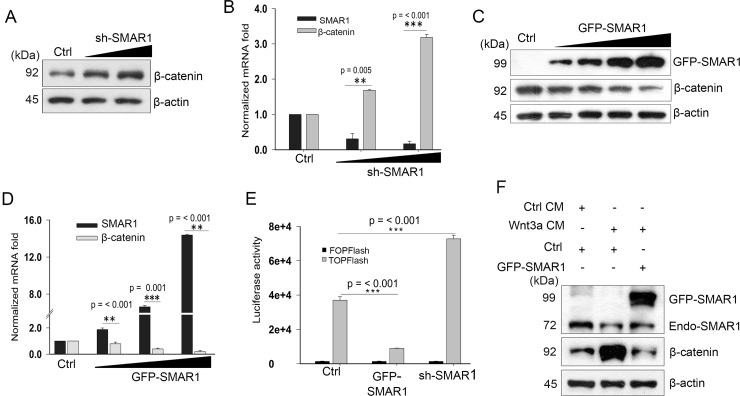
SMAR1 inhibits Wnt/β-catenin signaling pathway (**A**) β-catenin expression in HCT116 cells after knockdown with sh-SMAR1. The triangle indicates increased concentration of SMAR1 plasmids of 1 and 3 μg. (**B**) Real-Time PCR experiment showing β-catenin mRNA levels in SMAR1 knockdown HCT116 cells. 18S rRNA was used for normalization. Results are shown as mean ± SD (*n* = 3). The *p* value was determined by student's *t*-test. The triangle indicates increased concentration of SMAR1 plasmids of 2 and 4 μg. (**C**) β-catenin expressions in HCT116 cells after GFP-SMAR1 overexpression. The triangle indicates increased concentration of SMAR1 plasmids of 1, 2, 3 and 4 μg. (**D**) Real Time-PCR experiment showing β-catenin mRNA levels in SMAR1 overexpressed HCT116 cells. 18s rRNA was used for normalization. Results are shown as mean ± SD (*n* = 3). The *p* value was determined by student's *t*-test. The triangle indicates increased concentration of SMAR1 plasmids of 1, 2.5 and 4 μg. (**E**) Luciferase promoter activities of Super 8X TOPFlash/FOPFlash (mean ± SD, *n* = 3) after SMAR1 overexpression and knockdown in HCT116 cells. (**F**) Expression of β-catenin in HCT116 cells after transfection with GFP-SMAR1 and simultaneous stimulation with Wnt3a CM.

After validating the correlation between Wnt3a activation and SMAR1 proteasomal degradation, we continued to study how SMAR1 stability restrained β-catenin expression. β-catenin is the main transcriptional co-activator of Wnt/β-catenin pathway [24] and so we monitored its expression upon knockdown and overexpressed conditions of SMAR1. We observed a significant increase in β-catenin protein (2.16-fold) and mRNA (3.18-fold) levels upon knockdown of SMAR1 (Figure 2A, 2B and Supplementary Figure 2A). In a reciprocal experiment, we observed that GFP-SMAR1 overexpression consistently downregulated β-catenin both at protein (3.21-fold) and mRNA (5.26-fold) levels (Figure 2C, 2D and Supplementary Figure 2B). Additionally, SMAR1 overexpression also downregulated β-catenin in various other CRC cell lines (Supplementary Figure 2C–2F).

Further analysis revealed that SMAR1 overexpression led to Wnt3a dependent TCF4/LEF1 transcriptional inhibition by reducing the association of β-catenin and LEF1 (Supplementary Figure 2G). The inhibition of Wnt/β-catenin activity was confirmed by the luciferase promoter activities of Super 8X TOPFlash and FOPFlash constructs, which contain the native and mutated TCF/LEF binding sites for β-catenin. Reduced TOPFlash luciferase promoter activities were observed upon overexpression with SMAR1; conversely, SMAR1 knockdown enhanced the luciferase promoter activities (Figure 2E). Further analysis revealed that Wnt3a stimulation failed to restore β-catenin levels concomitant with SMAR1 overexpression (Figure 2F). These findings indicate that SMAR1 suppresses β-catenin mRNA and restricts the Wnt/β-catenin signaling activities.

### SMAR1 suppresses β-catenin promoter activities

**Figure 3:**
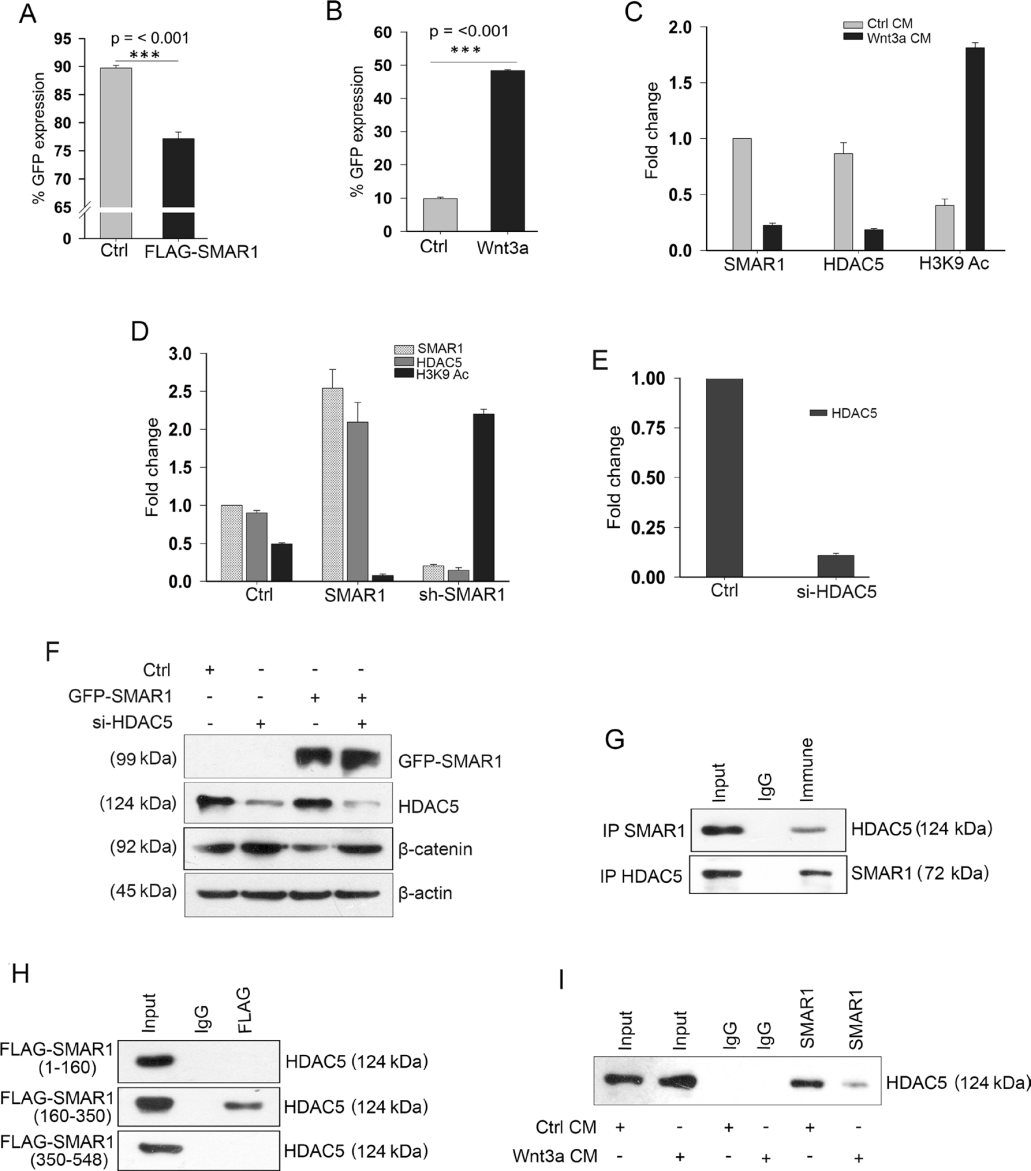
SMAR1 suppresses β-catenin promoter activities FACS analysis of pEGFP1-β-catenin GFP expression (*n* = 3, SD) after; (**A**) Co-transfection with FLAG-vector or FLAG-SMAR1, and (**B**) Treatment with 200 ng/mL rh Wnt3a ligand. (**C**) ChIP showing occupancy of SMAR1, HDAC5 and H3K9 Ac (mean ± SD, *n* = 3) after Wnt3a CM stimulation. (**D**) ChIP showing occupancy of SMAR1, HDAC5 and H3K9 Ac (mean ± SD, *n* = 3) after SMAR1 overexpression or knockdown. (**E**) ChIP showing occupancy of HDAC5 (mean ± SD, *n* = 3) after si-HDAC5 knockdown in HCT116 cells. (**F**) Expression of β-catenin after knockdown with 2μg si-HDAC5 plasmid. (**G**) Immunoprecipitation of SMAR1 with HDAC5. (**H**) Immunoprecipitation of HDAC5 with various truncations of SMAR1. (**I**) Immunoprecipitation of HDAC5 with SMAR1 after Wnt3a CM stimulation.

To study SMAR1 mediated transcriptional inhibition of β-catenin, we searched for putative Matrix-Attachment Region (MAR) sequences in the β-catenin promoter. MARs are generally AT-rich DNA sequence that spans from 50–500 bases in length and attaches to the nuclear matrix [25]. These MAR sequences also harbor various transcription factor binding sites [26]. Using MAR-Wiz software that analyse the AT-rich pattern [27] a significant MAR potential region was observed at −1950 to −2100 bases before the transcription start site (Supplementary Figure 3A and 3B). The MAR site predicts the probable SMAR1 occupancy in the β-catenin promoter. A chromatin immunoprecipitation (ChIP) experiment was performed using SMAR1 antibody and amplified the eluted DNA using primers spanning the predicted SMAR1 binding site. The ChIP-PCR experiment demonstrated specific amplification in the immune pulled-out fraction, confirming SMAR1 occupancy at β-catenin promoter (Supplementary Figure 3C). Seeking additional data to monitor the promoter activity we cloned the β-catenin promoter having SMAR1 binding sequences into pEGFP1 vector and christened as pEGFP1-β-catenin. Using flow cytometry technique we quantitated the GFP expression that directly correlated with the β-catenin promoter activities. In a co-transfection experiment with pEGFP1-β-catenin, we observed a significant decrease in the GFP expression (~15%) upon FLAG-SMAR1 overexpression in comparison to FLAG-control (Figure 3A and Supplementary Figure 3D). A significant increase of about 45% in GFP expression was observed when pEGFP1-β-catenin transfected HCT116 cells were treated using 200 ng/mL rh Wnt3a ligand (Figure 3B and Supplementary Figure 3E). Taken together, these observations confirm the important inhibitory role played by SMAR1 occupancy in the β-catenin promoter that accounts for the transcription silencing of β-catenin in CRC.

Earlier studies demonstrated that SMAR1 serves as a docking site for several chromatin modifiers that modulate the chromatin architecture for transcription inhibition of target genes [14]. An increased occupancy of H3K9 Acetylation (Ac) has been observed in β-catenin promoter in absence of HDAC5, which accounts for the transcriptional activation of β-catenin [28]. These reports prompted us to look into SMAR1-mediated histone modifications in the β-catenin promoter. We observed an increased H3K9 Ac (4.72-fold) occupancy but reduced occupancy of SMAR1 (4.54-fold) and HDAC5 (4.77-fold) in the promoter region upon Wnt3a stimulation (Figure 3C). A stable knockdown of SMAR1 further amplifies H3K9 Ac occupancy (4.48-fold) in the β-catenin promoter, thus upregulating Wnt signaling in CRCs (Figure 3D and Supplementary Figure 3F). In a reciprocal experiment overexpression of SMAR1 led to increased HDAC5 (2.22-fold) occupancy at β-catenin promoter that causes localized deacetylation and transcriptional silencing of β-catenin (Figure 3D and Supplementary Figure 3G). Functional specificity of HDAC5 was confirmed as SMAR1 fails to suppress β-catenin in the absence of HDAC5 recruitment at β-catenin promoter (Figure 3E and 3F). Since enrichment of HDAC5 was observed, therefore we further investigated whether SMAR1 interacts with HDAC5 and truncated SMAR1 into different fragments *viz*, FLAG-SMAR1 (1–160), FLAG-SMAR1 (160–350) and FLAG-SMAR1 (350–548) amino acids to identify specific SMAR1 domain for HDAC5 interaction. We found that SMAR1 interacts with HDAC5 and specifically FLAG-SMAR1 (160–350) fragment participates in the interaction with HDAC5 (Figure 3G and 3H). Upon Wnt3a activation, this interaction of SMAR1 with HDAC5 is reduced (Figure 3I). The consequence of reduced occupancy of SMAR1 and HDAC5 resulted in an enhanced H3K9 Ac enrichment in β-catenin promoter. These results suggest that SMAR1 is crucial in recruiting HDAC5 that deacetylates β-catenin promoter to inhibit the transcription.

### SMAR1 inhibits CRC cell migration and invasion

**Figure 4:**
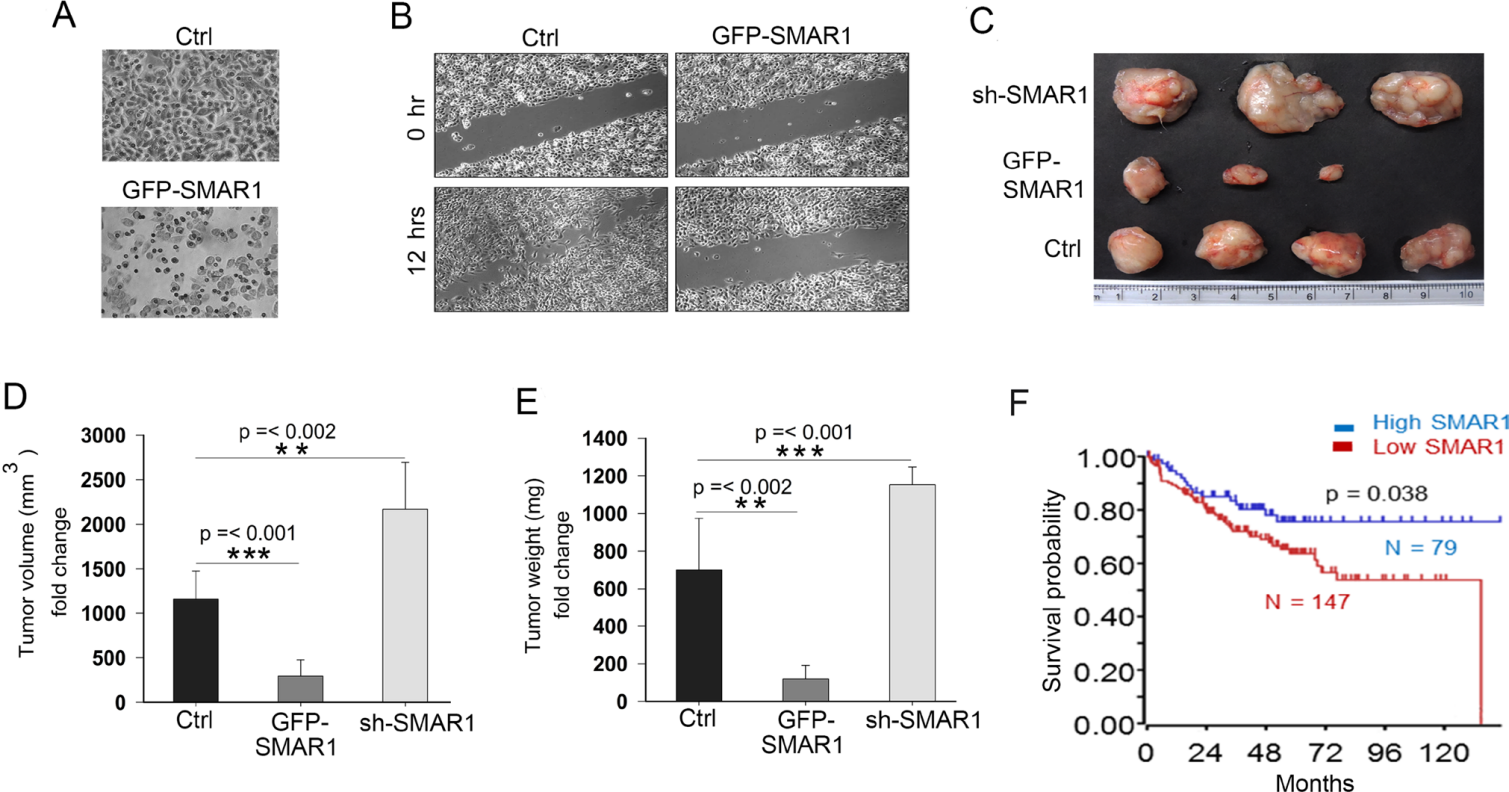
SMAR1 inhibits CRC cell migration and invasion (**A**) Cell invasion in SW480 cells after overexpression with GFP-SMAR1 construct. (**B**) Cell migration assay in GFP-SMAR1 overexpressed SW480 cells. (**C**) Tumors generated in NOD-SCID mice (*n* = 10) using various stable HCT116 cells for SMAR1. (**D** and **E**) Graphs showing volume and weight of tumors generated in NOD-SCID mice. (**F**) Kaplan Meier survival probability curve plotted with respect to SMAR1 expression in CRC patients. The survival curve was generated using Smith tumor colon database.

The attenuation of Wnt/β-catenin activities by SMAR1 further supports the translational value of SMAR1 in CRC. Therefore we sought to determine the tumor suppressive properties of SMAR1. Overexpression of SMAR1 in SW480 cells significantly reduced the number of matrigel invading cells as compared to the control (Figure 4A). To show the anti-metastatic properties of SMAR1, metastatic SW480 cells were overexpressed using GFP-SMAR1 construct and a wound healing assay was performed. After 12 hours of the wound generation, it is observed that SMAR1 overexpressed cells significantly retarded the migration of SW480 cells (Figure 4B). Additionally, we analysed the anti-tumor properties of SMAR1 in *in-vivo* mice model. Stable HCT116 CRC cells were generated using GFP-SMAR1 and GFP-sh-SMAR1 constructs to raise tumors in NOD-SCID mice. These stable cells were subcutaneously injected into the mice to raise tumors. Results show that the mice group injected with GFP-SMAR1 stable cells generates significantly smaller tumor sizes as compared to the control and GFP-sh-SMAR1 stable cells injected mice group (Figure 4C–4E). These results suggest that SMAR1 suppresses CRCs both in *in-vitro* and *in-vivo* models. The importance of SMAR1 levels are also reflected in CRC patients with respect to patient survival. The Kaplan Meier survival probability curve (https://hgserver1.amc.nl/cgi-bin/r2/main.cgi) shows higher death in CRC patients associated with low SMAR1 levels (Figure 4F). Collectively, these results suggest the significance of SMAR1 as a tumor suppressor in CRC.

### Microbial peptides attenuate Wnt/β-catenin signaling

Our current research highlights that SMAR1 is a substrate in the Wnt3a driven proteasomal degradation and restoration of SMAR1 inhibits tumor growth by down-modulating Wnt/β-catenin signaling activities. Accordingly, prevention of SMAR1 proteolysis may hold great potential in the development of novel CRC therapeutic approaches. We reasoned that if the D-box elements are no longer accessible, SMAR1 degradation would be sufficiently retarded, promoting its constitutive stability in CRC. Several studies have highlighted the use of peptides for blocking the protein-protein interactions [29, 30]. Since, microbial peptides demonstrate the promise of functioning as highly selective, efficient and yet non-toxic oncology therapeutics [31], therefore we have focused here on novel microbial peptides.

**Figure 5:**
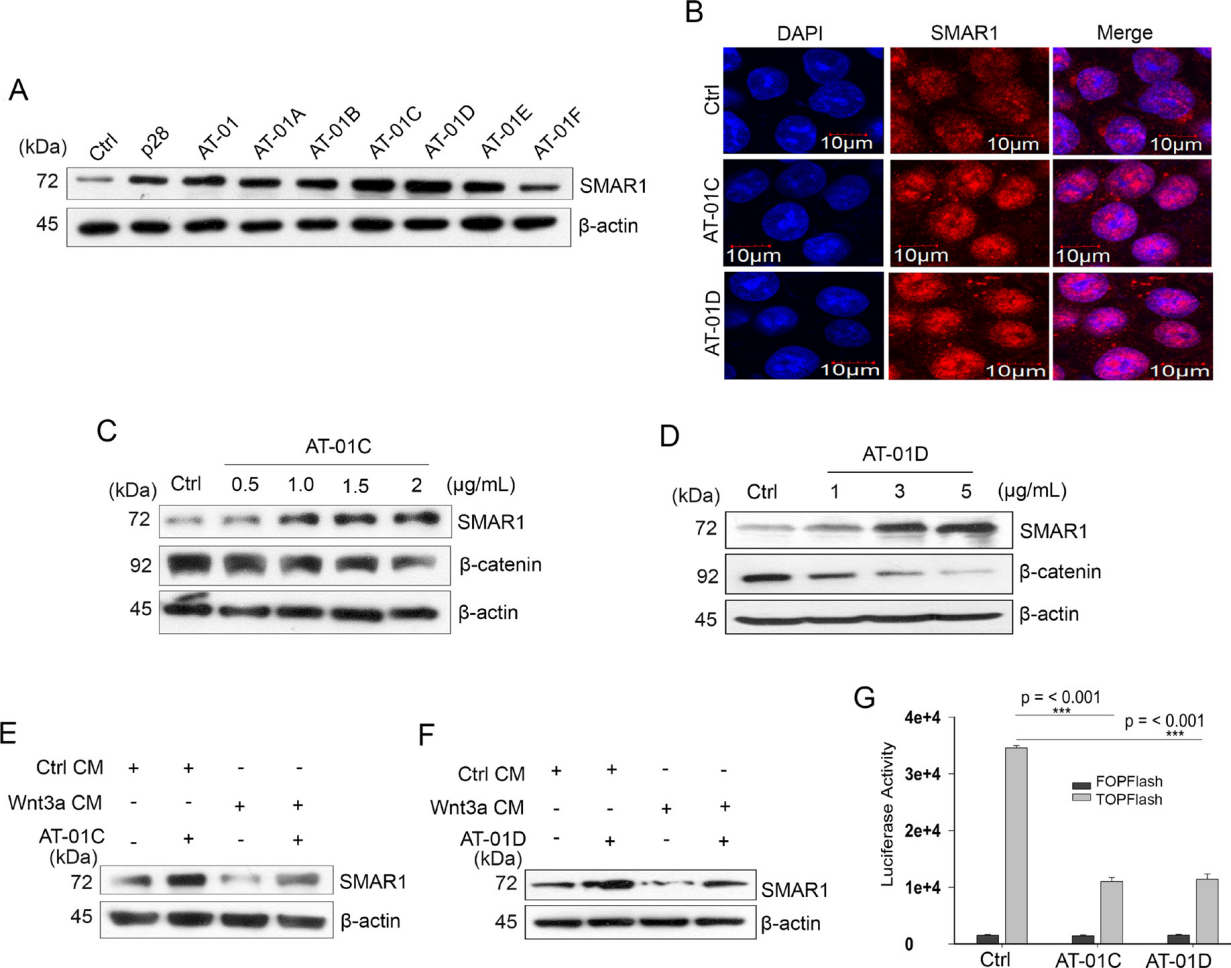
Microbial peptides attenuate Wnt/β-catenin signaling (**A**) Expression of SMAR1 in HCT116 cells treated with various peptides (5 μg/mL) for 48 hrs. (**B**) Confocal staining for SMAR1 after AT-01C or AT-01D (10 μg/mL) treatment. The scale bar used in the confocal experiment represents 10 μm. β-catenin expression after treatment of HCT116 cells with increasing concentration of; (**C**) AT-01C and (**D**) AT-01D peptides. SMAR1 expression after stimulation of HCT116 cells with Wnt3a CM and simultaneously treated with (**E**) AT-01C, and (**F**) AT-01D. (**G**) Luciferase promoter activities of Super 8X TOPFlash/FOPFlash (mean ± SD, *n* = 3) after treatment with AT-01C or AT-01D.

For our study we considered the role of microbial peptides to protect SMAR1 proteasomal degradation by masking the D-box elements. We focused on a 30 amino acid long peptide AT-01 derived from MPT63 (16 kDa), a secretory protein of *M. tuberculosis* [32] (Supplementary Figure 4A). We further studied six modified and truncated peptides of AT-01 viz, AT-01A to AT-01F in support of our hypothesis (Table 1 and Supplementary Figure 4B–4H). Upon treatment in an array of CRC cell lines, we observed the highest potential in peptides AT-01C and AT-01D in terms of stabilizing SMAR1 expression (Figure 5A, 5B and Supplementary Figure 4I and 4J). However, the SMAR1 mRNA levels remain unaltered suggesting the ability of these peptides to stabilize SMAR1 protein (Supplementary Figure 4K and 4L). These peptides also downregulated β-catenin levels (Figure 5C and 5D) and are able to restore SMAR1 expressions despite Wnt3a stimulation (Figure 5E and 5F). MTT assay shows that the IC_50_ value of AT-01C and AT-01D is 10 μg/mL and 9.90 μg/mL respectively in HCT116 cells (Supplementary Figure 4M and 4N). Furthermore, AT-01C and AT-01D inhibited the Super 8X TOPFlash luciferase promoter activities (3.5-fold) suggesting their ability to attenuate Wnt/β-catenin signaling activities (Figure 5G). Taken together, our data shows that the microbial peptides AT-01C and AT-01D prevent Wnt3a mediated proteasomal degradation of SMAR1 and thereby downregulates β-catenin expression.

**Table 1:** List of peptides derived from MPT63

Peptide	Amino acid sequence	Mol. Wt.	No. of residues
AT-01	GQVWEATATVNAIRGSVTPAVSQFNARTAD	3.30 kDa	30
AT-01A	ATATVNAIRGSVTPAVSQFNARTAD	2.75 kDa	25
AT-01B	GQVWEATATVNAIRGSVTPAVSQFN	2.75 kDa	25
AT-01C	ATATVNAIRGSVTPAVSQFN	2.20 kDa	20
AT-01D	NAIRGSVTPAVSQFN	1.65 kDa	15
AT-01E	ATATVNAIRGSVTPA	1.65 kDa	15
AT-01F	NAIRGSVTPA	1.10 kDa	15

List of peptides derived from MPT63. AT-01 peptide was truncated and further generated 6 peptides. The name of the peptides, amino acid sequences, molecular weights and the number of amino acid residues are shown.

### AT-01C and AT-01D mask D-box elements of SMAR1

**Figure 6:**
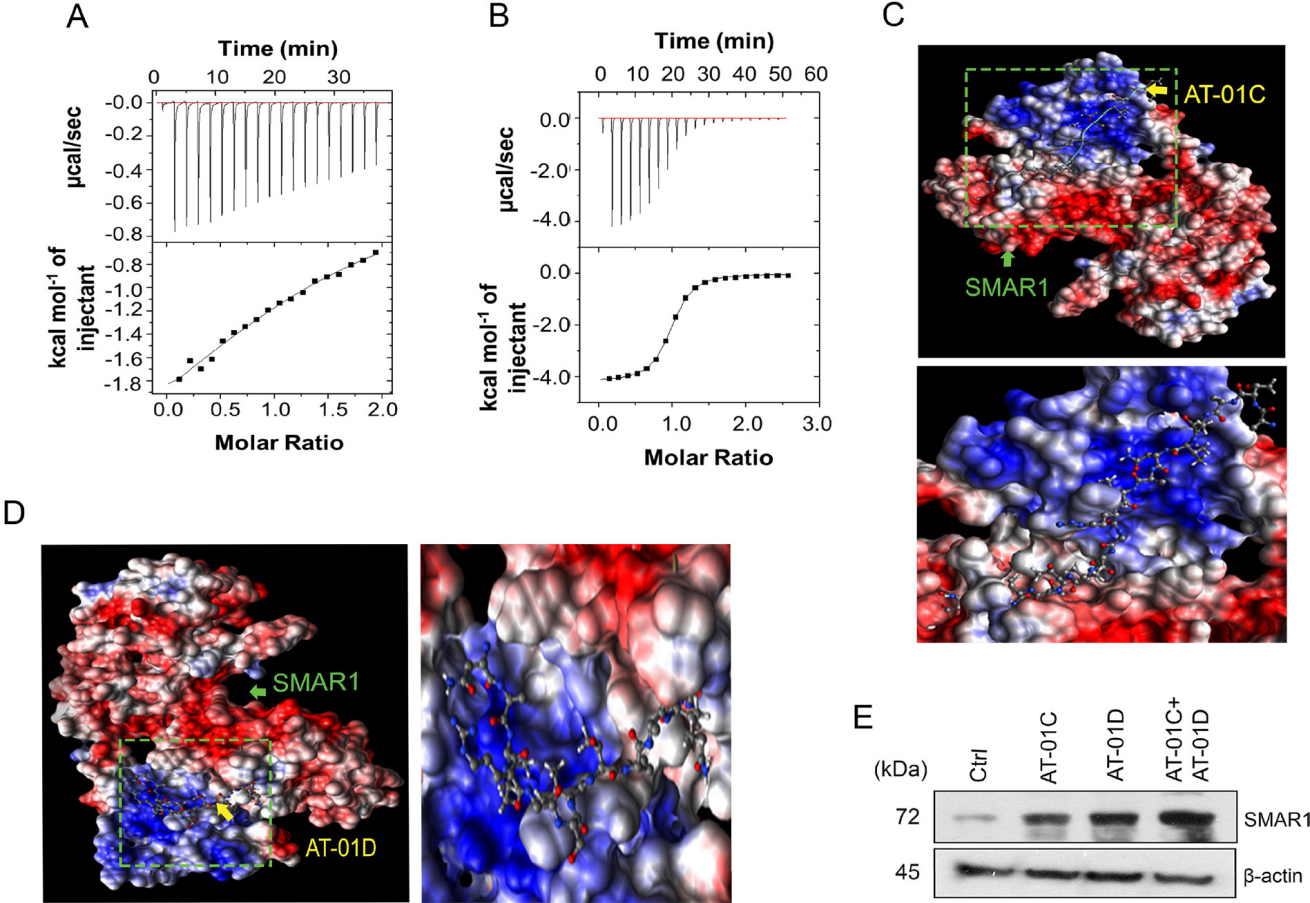
AT-01C and AT-01D mask D-box elements of SMAR1 Isothermal Titration Calorimetry (ITC) showing the interaction of SMAR1 with the peptides, (**A**) AT-01C and (**B**) AT-01D. *In silico* AutoDock interactions of SMAR1 with: (**C**) AT-01C and (**D**) AT-01D peptide. (**E**) SMAR1 expression after treating HCT116 cells with single peptide or combination of AT-01C and AT-01D.

To delineate the precise mechanism of SMAR1 protein stability, we studied the *in-vitro* interaction profiles of SMAR1 with AT-01C and AT-01D. Isothermal Titration Calorimetric (ITC) analysis has been extensively used to predict and confirm the interaction of peptides with proteins [33]. SMAR1 protein was subjected to ITC analysis with AT-01C and AT-01D peptides to examine their interactions. The graphs from the ITC profile revealed that both AT-01C and AT-01D interacts with SMAR1 (Figure 6A and 6B). The ΔG (kcal) values from ITC analysis predicted favourable interaction of SMAR1 with the peptides (Table 2).

**Table 2:** ΔG (kcal) values of the ITC analysis

Peptides	Interacting partner	ΔG
AT-01C	SMAR1	−3.984
AT-01D	SMAR1	−6.919

ΔG (kcal) values of the ITC analysis. ΔG (kcal) values resulted from the ITC analysis predicted favourable interactions of SMAR1 with AT-01C and AT-01D. The ΔG values resulted from the ITC experiment was performed at room-temperature.

*In silico* docking analysis using AutoDock [34] too demonstrated the interaction of SMAR1 with AT-01C and AT-01D (Figure 6C and 6D). PDBsum analysis [35, 36] revealed that AT-01C binds partially to the “RCHL” *via* Arginine, Cysteine and along with other amino acid residues in blue font (Supplementary Figures 5A and 1F). On the other hand AT-01D binds to the portion of “RQRL” at Glutamine, Arginine, Leucine and along with other amino acid residues in red font (Supplementary Figures 5B and 1F). Thus, AT-01C and AT-01D binds separately and perturb the function of the D-box elements from initiating the proteasomal degradation of SMAR1. Hence, it is highly likely that the combination of AT-01C and AT-01D would further stabilize SMAR1 by blocking “RQRL” and “RCHL” respectively and simultaneously (Figure 6E). These results suggest that the peptides bind to the D-box elements and protect SMAR1 from proteasomal degradation.

### AT-01C and AT-01D exhibit anti-cancer potential

**Figure 7:**
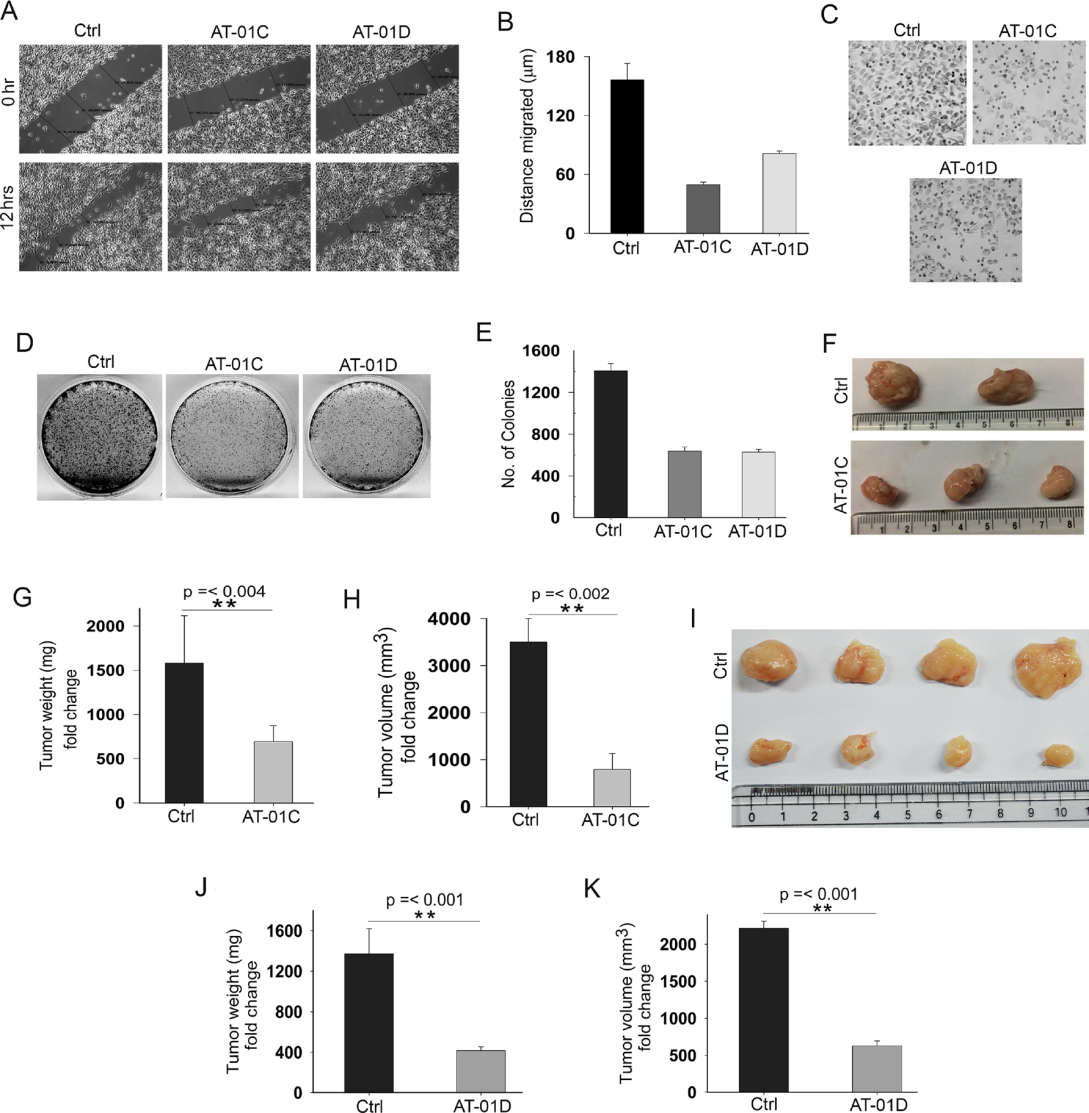
AT-01C and AT-01D exhibit anti-cancer potential (**A**) Cell migration assay in SW480 cells treated with AT-01C or AT-01D for 12 hours. (**B**) Graphical representation of SW480 cells migrated (mean ± SD, distance in triplicates arbitrarily taken at three different points). (**C**) Cell invasion assays in SW480 cells after treatment with AT-01C or AT-01D (10 μg/mL) for 48 hrs. (**D**) Clonogenic assays in HCT116 cells after treatment with AT-01C or AT-01D (10 μg/mL), and (**E**) Its graphical representation (*n* = 3, SD). (**F**) Tumors generated using 1 × 10^6^ HCT116 cells in NOD-SCID mice after administration with AT-01C (25 mg/kg body weight). (**G** and **H**) Graph representing the weight and volume of the mice tumors administered with AT-01C. (**I**) Tumors generated using 1 × 10^6^ HCT116 cells in NOD-SCID mice after administration with AT-01D (25 mg/kg body weight). (**J** and **K**) Graph representing the weight and volume of the mice tumors administered with AT-01D.

Recent studies provide evidences that increased cell metastasis in cancer cells are closely correlated with higher β-catenin activities [37] and reduced SMAR1 expression [12]. Accordingly, we carried out a wound healing assay to investigate the anti-metastatic potential of AT-01C and AT-01D microbial peptides. Substantially decreased cell migrations were observed when metastatic SW480 cells were treated with AT-01C (3-fold reduction) and AT-01D (2.14-fold reduction) as compared to the untreated cells (Figure 7A and 7B). Further, the cell invasion assay revealed the matrigel anti-invasive potential of AT-01C and AT-01D peptides in SW480 cells (Figure 7C). To analyze the anti-proliferative potential of these peptides a colony formation assay was performed, where AT-01C and AT-01D treated cells showed significantly lesser colony numbers (Figure 7D). More specifically, we observed a 2.33-fold decrease in the number of colonies in both AT-01C and AT-01D treated HCT116 cells as compared to the control (Figure 7E). Accordingly, these microbial derived peptides clearly demonstrate the tumor suppressive effects in *in-vitro* CRC cells.

Continuing our studies of the AT-01C and AT-01D peptides as anti-tumor in *in-vivo* mouse model, we raised subcutaneous tumors as detailed in Materials and Methods in NOD-SCID mice. The tumors were generated using HCT116 cells for AT-01C and AT-01D peptide administration. Peptides (25 mg/kg body weight of mice) were intra-peritoneally administered after the tumors became visible. Interestingly, reduction in tumor weight (2.5-fold) and volume (3.88-fold) was observed in the mice group that received the AT-01C peptide (Figure 7F–7H). Similarly, we also observed reduction in tumor weight (3.29-fold) and volume (3.52-fold) for mice group that received AT-01D peptide (Figure 7I–7K). Further, there were no observable adverse effects in the peptide administered mice throughout the course of treatment, supporting the non-toxic nature of the microbial peptides. These outcomes suggest substantial therapeutic potential of AT-01C and AT-01D as novel CRC therapies, in conjunction with the SMAR1 stability.

## DISCUSSION

Earlier studies have shown that aberrant Wnt/β-catenin signaling activities remain predominately upregulated in CRCs that translates into a constitutive activation of β-catenin [38]. In addition, reduced SMAR1 expression is also frequently associated with various human cancers leading to poor prognosis of the diseases [7]. Enhanced lung metastasis has been reported upon SMAR1 knockdown in breast cancers by an increased alternative splicing of CD44. In another report it is notable that reduced expression of SMAR1 in breast cancers leads to increase cell proliferation, invasion and metastasis. In fact, in several types of cancers loss of heterozygosity of SMAR1 gene in 16q24.3 arm of the chromosome leads to decreased SMAR1 expression [7]. A recent study suggests that Cell Division Cycle 20 (CDC20) is involved in proteasomal degradation of SMAR1 [22]. Now our investigation demonstrates that Wnt3a activation mediates proteasomal degradation of SMAR1 (Figure 1G). But the involvement of any ubiquitin ligases leading to the Wnt3a mediated proteasomal degradation of SMAR1 need to be studied further. Accordingly, there may be therapeutic advantage targeting SMAR1 to amplify its tumor suppressive effects.

The tumor suppressor potential of SMAR1 will be evident if Wnt/β-catenin signaling activities are significantly attenuated in CRCs. We note that SMAR1 overexpression blocks Wnt3a mediated stabilization of β-catenin by inhibiting the transcription (Figure 2C and 2F). Tumor suppressors like Axin, APC etc. has been reported to govern proteasomal degradation of β-catenin and thus attenuates Wnt/β-catenin activities. However, mutation in the phosphorylation sites of β-catenin at its N-terminus negates the suppressive function of these tumor suppressors [39, 40]. Thus, targeting the mRNA of β-catenin may mitigate the challenge of gene mutations such as in the phosphorylation sites of β-catenin. The transcription inhibition of β-catenin is attributed to SMAR1 occupancy in the β-catenin promoter (Figure 3D). A pEGFP1-β-catenin promoter cloned encompassing putative SMAR1 binding site reveals inhibition of promoter activities in the presence of SMAR1 (Figure 3A). pEGFP1-β-catenin promoter also responded to Wnt3a stimulation and increased β-catenin promoter activities suggesting that the promoter region to which SMAR1 binds is crucial to Wnt3a signaling (Figure 3B). An earlier report suggests the importance of HDAC5 recruitment in the β-catenin promoter that represses the transcription of β-catenin [28]. Here, we showed that presence of SMAR1 is crucial in recruiting HDAC5 and maintains a deacetylate state that allows chromatin condensation preventing H3K9 Ac occupancy in the β-catenin promoter (Figure 3D). However, the recruitment of HDAC5 drastically decreases during Wnt3a stimulation in the β-catenin promoter that further favors H3K9 Ac recruitment and β-catenin transcription (Figure 3C). Aberrant Wnt3a signaling cause proteasomal degradation of SMAR1 and subsequently fails to recruit HDAC5 to the β-catenin promoter. Therefore, SMAR1 is crucial in maintaining the transcriptional silencing of β-catenin. The role of SMAR1 in attenuating Wnt/β-catenin activities is also confirmed from the decreased Super 8X TOPFlash luciferase promoter activities during SMAR1 overexpression (Figure 2E). Thus, SMAR1 negatively regulates Wnt/β-catenin activities and provides a new pathway for more effective suppression of Wnt/β-catenin signaling as therapeutics of CRC.

Tumor suppressor proteins have been reported to undergo proteasomal degradation that results in increased tumorigenesis [41]. Thus, it is crucial to prevent ubiquitin degradation of such proteins to enhance the tumor suppressive effect [42]. In this context, we have shown that stabilization of SMAR1 inversely controls Wnt/β-catenin activities thus resulting into reduced cell migration and invasion of CRC in *in-vitro* as well as *in-vivo* (Figure 4A–4E). Understanding that SMAR1 negatively regulates β-catenin, the approach to target SMAR1 also should effectuate downregulation of Wnt signaling in CRCs. Recent reports suggest that peptides can be effectively used to target and block ubiquitin ligase sites and have been perceived as therapeutic agents [43]. Moreover, microbial peptides such as p28 derived from Azurin, a secretory protein of *Pseudomonas aeruginosa* have been successfully demonstrated to stabilize p53 protein that inhibits cancer cell growth [44]. *Bacille Calmette-Guérin* (BCG), the causative agent of tuberculosis has been reported to be utilized in early immune-oncology therapy and remaining today the primary therapy for bladder cancer [45]. Therefore, we selected microbial peptides that would selectively target and stabilize SMAR1. Because SMAR1 undergoes proteasomal degradations, therefore these peptides have to be selected such that D-box elements are masked. Consequently, Wnt3a stimulation will fail to proteasomally degrade SMAR1. We have used peptides from MPT63, a secretory protein of *Mycobacterium tuberculosis* for our studies [46]. Amongst the many peptides of MPT63, the AT-01C and AT-01D peptide treated cells displayed the most elevated expression of SMAR1 that led us forward to analyze the functional mechanism (Figure 5A). From the isothermal titration calorimetric [33] and docking studies [34] it is revealed that SMAR1 interacts with these two peptides (Figure 6A–6D). Detailed analysis of the docked protein-peptide interactions using PDBsum [35, 36] revealed that these peptides target the D-box elements in SMAR1. Because Arginine and Leucine are important for retaining the D-box functions [22] therefore, partial masking of the “RQRL” or “RCHL” blocked the proteasomal degradation of SMAR1. Based on our experiments it is evident that substitution mutation of only one of the D-box elements let to lesser SMAR1 stabilization as compared to mutating both the D-box elements (Figure 1H). Because either treatment of AT-01C or AT-01D peptides individually could block only one D-box at a time, therefore we concluded that concurrent treatment with both peptides would prevent any proteasomal degradation of SMAR1 (Figure 6E). Further, stabilized SMAR1 expression due to the peptide treatments suppressed the expression of β-catenin (Figure 5C and 5D) and Super 8X TOPFlash luciferase promoter activities that confirmed the potential to block Wnt/β-catenin signaling (Figure 5G). Consequently, these peptides significantly perturbed cell proliferation, invasion and metastasis in *in-vitro* and tumor size reductions in *in-vivo* (Figure 7A–7K).

This study reports the significance of SMAR1 as a tumor suppressor and reveals the mechanism of SMAR1 on cell migration and invasion. SMAR1 negatively regulates Wnt/β-catenin signaling and further inhibits cellular metastatic activities both *in-vitro* and *in-vivo*. As Wnt/β-catenin activities have been reported to be involved in maintaining cell metastasis [47], it has become a promising target of clinical importance. The microbial peptides that targets SMAR1 stabilization to further attenuate Wnt/β-catenin activities may be used as novel CRC therapeutics.

## MATERIALS AND METHODS

### Cell culture, peptide treatment and transfection

HCT116, SW480, SW620, HCT15 and HT29 cells (obtained from NCCS, Pune) were cultured in DMEM medium with 10% Fetal Bovine Serum (Gibco) and 100 Units of Penicillin Streptomycin antibiotics (Gibco). HCT116 stable cells were generated using GFP-SMAR1, GFP-control and GFP-sh-SMAR1 plasmids. si-HDAC5 plasmid was purchased from Santa Cruz Biotechnology. Conditioned medium were prepared using L cells and L-Wnt3a cells (kind gift from Jomon Joseph, NCCS Pune). After culturing for 7 days supernatant was collected and filtered through 0.22 micron syringe filter. Cells were treated with peptides (provided by Amrita Therapeutics) for 48 hours. Lipofectamine 2000 (Invitrogen) was used for transfection purposes.

### Luciferase assay

HCT116 cells were co-transfected with GFP-SMAR1 or GFP-control together with Super 8× TOPFlash/FOPFlash plasmids (kind gift from Nibedita Lenka, NCCS). After 48 hours, proteins were extracted using Passive Lysis Buffer (Promega). For luciferase assays 100 μg proteins were calculated and PBS was added to make the volume up to 100 μl. 100 μl of 1× Luciferase assay reagent (Promega) was added to the protein lysates and the luminescence reading was recorded using GloMax 96 Microplate Luminometer. The graph showing the promoter activities of Super 8× TOPFlash/FOPFlash was generated using SigmaPlot software.

### Cell migration, matrigel invasion and clonogenic assay

For cell migration SW480 cells were either transfected with GFP-SMAR1 or treated with microbial peptides AT-01C and AT-01D (10 μg/mL). Wounds were created and images of the migrations were acquired in a confocal microscope (Nikon). For matrigel invasion assays SW480 cells were either transfected with GFP-SMAR1 or treated with AT-01C and AT-01D (10 μg/mL) and then seeded in matrigel chambers. The matrigel invaded cells were fixed using methanol and washed using PBS buffer. Cells were stained with 0.1% crystal violet (Sigma-Aldrich) solution. For clonogenic assays HCT116 cells were treated with AT-01C and AT-01D (10 μg/mL) every 24 hours for 14 days. Cells were fixed using 1% formaldehyde solution (Sigma-Aldrich) and stained using 0.1% crystal violet solution, where 50 or more cells were counted as one colony.

### Cloning of β-catenin promoter

Genomic DNA was isolated from HCT116 cells and amplified with primers flanking binding sites of SMAR1 in β-catenin promoter. The forward primer 5′-TCTGGTACCAGAACCACTTGTCTGTCGCC-3′ encodes XhoI and the reverse primer 5′-AGACTCGAGAGTGTAGCTATTGGTTGTGGTC-3′ encodes KpnI restriction sites. The amplicon was ligated into pEGFP1 vector using T4 DNA ligase (NEB) after restriction digestion with XhoI and KpnI enzymes (NEB). Plasmids were transformed into DH5α *E. coli* competent cells and positive clones were identified after restriction digestion with XhoI and KpnI. The cloned β-catenin promoter was christened as pEGFP1-β-catenin.

### Western blot and immunoprecipitation (IP)

Proteins were extracted using lysis buffer (50 mM Tris-Cl pH 7.5, 5 mM EDTA, 0.5% NP40, 50 mM NaF, 1 mM DTT, 0.2 mM sodium orthovanadate, 0.5 mM PMSF, 150 mM NaCl and 1X Protease Inhibitor cocktail). 40 μg proteins were transferred to PVDF membranes by Western blotting technique and blots were blocked with 5% BSA and incubated in primary antibodies. Blots were then incubated with HRP conjugated secondary antibodies and developed in X-ray films. For IP, proteins extracted were pre-cleared with Protein A/G beads (Invitrogen) and immunoprecipitated overnight with desired antibodies. Protein A/G beads were used to pull-down the protein-antibody complex and western blot was performed to check the interaction.

### Confocal

For confocal studies cells were fixed with 4% para-formaldehyde solution and permeabilized using 0.1% TritonX-100 solution. Cells were incubated with 1:250 dilution SMAR1 (Bethyl) antibody for 2 hours and later with 1:250 dilution Cy3 (Life Technologies) antibody for 1 hour. Coverslips containing cells were mounted using DAPI containing mounting medium (Fluoroshield with DAPI, Sigma). Human colon paraffinized tissue sections were de-paraffinized by heating in a microwave oven and later washed with xylene and alcohol. Antigen retrieval was performed and washed with PBS for further staining with 1:250 SMAR1 (Bethyl) and 1:250 β-catenin (BD Bioscience) antibodies. Alexa 488 and Cy3 (Life Technologies) were used as secondary antibodies for SMAR1 and β-catenin respectively. Images were observed in a confocal microscope (Olympus).

### Chromatin immunoprecipitation (ChIP)

HCT116 cells were transfected with GFP-control, GFP-SMAR1, GFP-sh-SMAR1 or HDAC5 siRNA plasmids. ChIP was performed using Millipore reagents supplied according to the manufacturer's protocol. The protein-DNA complex were immunoprecipitated overnight at 4° C using SMAR1 (Bethyl), HDAC5 (Cell Signaling), H3K9Ac (Cell Signaling) or IgG (Santa Cruz Biotechnology) antibodies (1 μg each). PCR was performed with the primers flanking the binding sites of SMAR1 in β-catenin promoter. Forward primer for β-catenin promoter is 5′- TCTGGTACCAGAACCACTTGTCTGTCGCC-3′ and 5′-AGACTCGAGAGTGTAGCTA TTGGTTGTGGTC-3′ as reverse primer. The PCR conditions were 95° C for 1 minute, 55° C for 1 minute and 72° C for 1 minute for 35 cycles. The PCR amplicons obtained were run in 1% Agarose (Sigma) gel and documented.

### Semi-quantitative and real-time PCR

The mRNA was extracted from cells by lysing in Trizol (Sigma) according to the manufacturer′s protocol. 1 μg mRNA were used to prepare the cDNA using reverse transcriptase enzyme (Bangalore Genei) at 42° C for 60 mins and 72° C for 10 mins. The cDNA obtained was used for mRNA amplification using specific primers for β-catenin, SMAR1, 18S rRNA and GAPDH. Primers used for PCRs were β-catenin forward primer 5′-CATCTACACAGTTTGATGCTGCT-3′ and reverse primer 5′-GCAGTTTTGTCAGTTCAGGGA-3′, SMAR1 forward primer 5′-CTTGCGGTTGGATAGC ATTGA-3′ and reverse primer 5′-GCTGCTTGTTCGTGA CCAGAT-3′, 18S rRNA forward primer 5′-GCTT AATTTGACTCAACACGGGA-3′ and reverse primer 5′-AGCTATCAATCTGTCAATCCTGTC-3′ and GAPDH forward primer 5′-TGCACCACCAACTGCTTAGC-3′ and reverse primer 5′-GGCATGGACTGTGGTCATG AG-3′. Real-Time PCRs were performed using SYBR Green (Bio-Rad). The PCR conditions were 95° C for 45 seconds, 60° C for 30 seconds and 72° C for 45 seconds for 40 cycles.

### Isothermal titration calorimetry (ITC) and docking

Peptides were analyzed for interaction with SMAR1 protein using ITC (MicroCal iTC_200_) instrument. The interaction studies of SMAR1 with AT-01C and AT-01D were performed at room-temperature. Thermographs were generated using Origin software. ΔG (kcal) was calculated using the formula: ΔG = ΔH – TΔS where ΔG is Gibbs energy changes, ΔH is enthalpy changes, T is the absolute temperature (kelvin), and ΔS is the entropy changes. The interaction of SMAR1 with AT-01C and AT-01D was also analyzed by an in silico docking AutoDock. Amino acid residues involved in the interaction were analyzed using PDBsum. The structures of the peptides were generated using Swiss PDB [48] while the SMAR1 structure was used from the already reported structure earlier from our lab [8].

### Flow cytometry

HCT116 cells were co-transfected with FLAG-SMAR1 and pEGFP1-β-catenin plasmids or treated with rh Wnt3a ligand after transfection with pEGFP1-β-catenin. For flow cytometry analysis HCT116 cells were trypsinized using Trypsin-EDTA (Gibco) and washed thrice with PBS buffer. Cells were then passed through cell strainer (BD Falcon) and collected in flow cytometry tubes. GFP expressions were quantitated by subjecting 1 × 10^4^ cells to BD FACSCalibur flow cytometry. FL1 channel was used to sort and quantify the GFP cells. Using CellQuest Pro software the histogram plots were derived and the GFP percentages were calculated. The graphs corresponding to the GFP percentage were generated using SigmaPlot software.

### MTT assay

HCT116 cells (6 × 10^3^) were seeded in a 96 well plate and cultured until confluency was attained. Cells were treated with increasing doses of AT-01C or AT-01D and incubated for 48 hours. Post 48 hours medium was replaced and fresh medium was added. MTT reagent (0.5 μg/mL) was added to the cells and incubated for another 4 hours. Post 4 hours of incubation the medium was removed carefully. The formazan formed was dissolved using iso-propyl alcohol (Sigma Aldrich). The absorbance was measured in a spectrophotometer at 570 nm wavelength. MTT was performed in triplicates and the graphs were plotted using SigmaPlot software.

### *In-vivo* tumor generation

Mice were obtained from NCCS animal house facility and treated in accordance with all relevant rules and ethical procedures of the NCCS institutional ethical committee. Subcutaneous tumors were raised in NOD-SCID (6-weeks) mice using 1 × 10^6^ stable SMAR1 and sh-SMAR1 HCT116 cells. For peptide treatments, subcutaneous tumors developed in NOD-SCID mice were administered with AT-01C or AT-01D peptides (25 mg/kg body weight). For *in-vivo* testing, these peptides were administered intra-peritoneally on alternate days for 21 days. For polyp generation in colon BALB/c mice were administered with Azoxymethane (Sigma) at a dose of 10 mg/kg body weight and fed with 2% Dextran Sodium Sulphate water.

## SUPPLEMENTARY MATERIALS FIGURES



## References

[1] Myers EA, Feingold DL, Forde KA, Arnell T, Jang JH, Whelan RL (2013). Colorectal cancer in patients under 50 years of age: a retrospective analysis of two institutions’ experience. World J Gastroenterol.

[2] Li H, Myeroff L, Smiraglia D, Romero MF, Pretlow TP, Kasturi L, Lutterbaugh J, Rerko RM, Casey G, Issa JP, Willis J, Willson JK, Plass C, Markowitz SD (2003). SLC5A8, a sodium transporter, is a tumor suppressor gene silenced by methylation in human colon aberrant crypt foci and cancers. Proc Natl Acad Sci USA.

[3] Grady WM, Carethers JM (2008). Genomic and epigenetic instability in colorectal cancer pathogenesis. Gastroenterology.

[4] Polakis P (2012). Wnt signaling in cancer. Cold Spring Harb Perspect Biol.

[5] Jones S, Chen WD, Parmigiani G, Diehl F, Beerenwinkel N, Antal T, Traulsen A, Nowak MA, Siegel C, Velculescu VE, Kinzler KW, Vogelstein B, Willis J, Markowitz SD (2008). Comparative lesion sequencing provides insights into tumor evolution. Proc Natl Acad Sci USA.

[6] Kuipers EJ, Grady WM, Lieberman D, Seufferlein T, Sung JJ, Boelens PG, van de Velde CJ, Watanabe T (2015). Colorectal cancer. Nat Rev Dis Primers.

[7] Malonia SK, Sinha S, Lakshminarasimhan P, Singh K, Jalota-Badhwar A, Rampalli S, Kaul-Ghanekar R, Chattopadhyay S (2011). Gene regulation by SMAR1: role in cellular homeostasis and cancer. Biochim Biophys Acta.

[8] Nakka KK, Chaudhary N, Joshi S, Bhat J, Singh K, Chatterjee S, Malhotra R, De A, Santra MK, Dilworth FJ, Chattopadhyay S (2015). Nuclear matrix-associated protein SMAR1 regulates alternative splicing via HDAC6-mediated deacetylation of Sam68. Proc Natl Acad Sci USA.

[9] Kaul R, Mukherjee S, Ahmed F, Bhat MK, Chhipa R, Galande S, Chattopadhyay S (2003). Direct interaction with and activation of p53 by SMAR1 retards cell-cycle progression at G2/M phase and delays tumor growth in mice. Int J Cancer.

[10] Chakraborty S, Adhikary A, Mazumdar M, Mukherjee S, Bhattacharjee P, Guha D, Choudhuri T, Chattopadhyay S, Sa G, Sen A, Das T (2014). Capsaicin-induced activation of p53-SMAR1 auto-regulatory loop down-regulates VEGF in non-small cell lung cancer to restrain angiogenesis. PLoS One.

[11] Adhikary A, Chakraborty S, Mazumdar M, Ghosh S, Mukherjee S, Manna A, Mohanty S, Nakka KK, Joshi S, De A, Chattopadhyay S, Sa G, Das T (2014). Inhibition of epithelial to mesenchymal transition by E-cadherin up-regulation via repression of slug transcription and inhibition of E-cadherin degradation: dual role of scaffold/matrix attachment region-binding protein 1 (SMAR1) in breast cancer cells. J Biol Chem.

[12] Singh K, Mogare D, Giridharagopalan RO, Gogiraju R, Pande G, Chattopadhyay S (2007). p53 target gene SMAR1 is dysregulated in breast cancer: its role in cancer cell migration and invasion. PLoS One.

[13] Jalota-Badhwar A, Kaul-Ghanekar R, Mogare D, Boppana R, Paknikar KM, Chattopadhyay S (2007). SMAR1-derived P44 peptide retains its tumor suppressor function through modulation of p53. J Biol Chem.

[14] Pavithra L, Rampalli S, Sinha S, Sreenath K, Pestell RG, Chattopadhyay S (2007). Stabilization of SMAR1 mRNA by PGA2 involves a stem loop structure in the 5′ UTR. Nucleic Acids Res.

[15] Zhang X, Hao J (2015). Development of anticancer agents targeting the Wnt/β-catenin signaling. Am J Cancer Res.

[16] Li L, Sheng Y, Li W, Hu C, Mittal N, Tohyama K, Seba A, Zhao YY, Ozer H, Zhu T, Qian Z (2017). β-Catenin Is a Candidate Therapeutic Target for Myeloid Neoplasms with del(5q). Cancer Res.

[17] Qazi A, Pal J, Maitah M, Fulciniti M, Pelluru D, Nanjappa P, Lee S, Batchu RB, Prasad M, Bryant CS, Rajput S, Gryaznov S, Beer DG (2010). Anticancer activity of a broccoli derivative, sulforaphane, in barrett adenocarcinoma: potential use in chemoprevention and as adjuvant in chemotherapy. Transl Oncol.

[18] Dufour A, Sampson NS, Li J, Kuscu C, Rizzo RC, Deleon JL, Zhi J, Jaber N, Liu E, Zucker S, Cao J (2011). Small-molecule anticancer compounds selectively target the hemopexin domain of matrix metalloproteinase-9. Cancer Res.

[19] Fosgerau K, Hoffmann T (2015). Peptide therapeutics: current status and future directions. Drug Discov Today.

[20] Chakrabarty AM, Bernardes N, Fialho AM (2014). Bacterial proteins and peptides in cancer therapy: today and tomorrow. Bioengineered.

[21] Stambolic V, Ruel L, Woodgett JR (1996). Lithium inhibits glycogen synthase kinase-3 activity and mimics wingless signalling in intact cells. Curr Biol.

[22] Paul D, Ghorai S, Dinesh US, Shetty P, Chattopadhyay S, Santra MK (2017). Cdc20 directs proteasome-mediated degradation of the tumor suppressor SMAR1 in higher grades of cancer through the anaphase promoting complex. Cell Death Dis.

[23] Morgan DO (2013). The D box meets its match. Mol Cell.

[24] MacDonald BT, Tamai K, He X (2009). Wnt/β-catenin signaling: components, mechanisms, and diseases. Dev Cell.

[25] Arope S, Harraghy N, Pjanic M, Mermod N (2013). Molecular characterization of a human matrix attachment region epigenetic regulator. PLoS One.

[26] Boulikas T (1994). Transcription factor binding sites in the matrix attachment region (MAR) of the chicken alpha-globin gene. J Cell Biochem.

[27] Singh GB, Kramer JA, Krawetz SA (1997). Mathematical model to predict regions of chromatin attachment to the nuclear matrix. Nucleic Acids Res.

[28] Zhao JX, Yue WF, Zhu MJ, Du M (2011). AMP-activated protein kinase regulates β-catenin transcription via histone deacetylase 5. J Biol Chem.

[29] Chang YS, Graves B, Guerlavais V, Tovar C, Packman K, To KH, Olson KA, Kesavan K, Gangurde P, Mukherjee A, Baker T, Darlak K, Elkin C (2013). Stapled α-helical peptide drug development: a potent dual inhibitor of MDM2 and MDMX for p53-dependent cancer therapy. Proc Natl Acad Sci USA.

[30] Mund T, Lewis MJ, Maslen S, Pelham HR (2014). Peptide and small molecule inhibitors of HECT-type ubiquitin ligases. Proc Natl Acad Sci USA.

[31] Sarafraz-Yazdi E, Bowne WB, Adler V, Sookraj KA, Wu V, Shteyler V, Patel H, Oxbury W, Brandt-Rauf P, Zenilman ME, Michl J, Pincus MR (2010). Anticancer peptide PNC-27 adopts an HDM-2-binding conformation and kills cancer cells by binding to HDM-2 in their membranes. Proc Natl Acad Sci USA.

[32] Manca C, Lyashchenko K, Wiker HG, Usai D, Colangeli R, Gennaro ML (1997). Molecular cloning, purification, and serological characterization of MPT63, a novel antigen secreted by Mycobacterium tuberculosis. Infect Immun.

[33] Ladbury JE, Chowdhry BZ (1996). Sensing the heat: the application of isothermal titration calorimetry to thermodynamic studies of biomolecular interactions. Chem Biol.

[34] Hetényi C, van der Spoel D (2002). Efficient docking of peptides to proteins without prior knowledge of the binding site. Protein Sci.

[35] Laskowski RA (2001). PDBsum: summaries and analyses of PDB structures. Nucleic Acids Res.

[36] Laskowski RA, Hutchinson EG, Michie AD, Wallace AC, Jones ML, Thornton JM (1997). PDBsum: a Web-based database of summaries and analyses of all PDB structures. Trends Biochem Sci.

[37] Lemieux E, Cagnol S, Beaudry K, Carrier J, Rivard N (2015). Oncogenic KRAS signalling promotes the Wnt/β-catenin pathway through LRP6 in colorectal cancer. Oncogene.

[38] Truant SC, Gouyer VP, Leteurtre EA, Zerimech F, Huet GM, Pruvot FR (2008). E-cadherin and beta-catenin mRNA levels throughout colon cancer progression. J Surg Res.

[39] Wu ZQ, Brabletz T, Fearon E, Willis AL, Hu CY, Li XY, Weiss SJ (2012). Canonical Wnt suppressor, Axin2, promotes colon carcinoma oncogenic activity. Proc Natl Acad Sci USA.

[40] Morin PJ, Sparks AB, Korinek V, Barker N, Clevers H, Vogelstein B, Kinzler KW (1997). Activation of beta-catenin-Tcf signaling in colon cancer by mutations in beta-catenin or APC. Science.

[41] Ciechanover A, Shkedy D, Oren M, Bercovich B (1994). Degradation of the tumor suppressor protein p53 by the ubiquitin-mediated proteolytic system requires a novel species of ubiquitin-carrier protein, E2. J Biol Chem.

[42] Kitagawa K, Kotake Y, Kitagawa M (2009). Ubiquitin-mediated control of oncogene and tumor suppressor gene products. Cancer Sci.

[43] Huang X, Dixit VM (2016). Drugging the undruggables: exploring the ubiquitin system for drug development. Cell Res.

[44] Yamada T, Christov K, Shilkaitis A, Bratescu L, Green A, Santini S, Bizzarri AR, Cannistraro S, Gupta TK, Beattie CW (2013). p28, a first in class peptide inhibitor of cop1 binding to p53. Br J Cancer.

[45] Morales A (2017). BCG: A throwback from the stone age of vaccines opened the path for bladder cancer immunotherapy. Can J Urol.

[46] Gomez M, Johnson S, Gennaro ML (2000). Identification of secreted proteins of Mycobacterium tuberculosis by a bioinformatic approach. Infect Immun.

[47] Ormanns S, Neumann J, Horst D, Kirchner T, Jung A (2014). WNT signaling and distant metastasis in colon cancer through transcriptional activity of nuclear β-Catenin depend on active PI3K signaling. Oncotarget.

[48] Guex N, Peitsch MC, Schwede T (2009). Automated comparative protein structure modeling with SWISS-MODEL and Swiss-PdbViewer: a historical perspective. Electrophoresis.

