# Supplementary material for: SMAR1 inhibits Wnt/β-catenin signaling and prevents colorectal cancer progression
Source: Oncotarget. 2018 Apr 20;9(30):21322–36. doi: 10.18632/oncotarget.25093 (PMC5940383; doi:10.18632/oncotarget.25093)
Supplement: Supplementary file 1 [file oncotarget-09-21322-s001.pdf]

SMAR1 inhibits Wnt/β-catenin signaling and prevents colorectal cancer progression

SUPPLEMENTARY MATERIALS

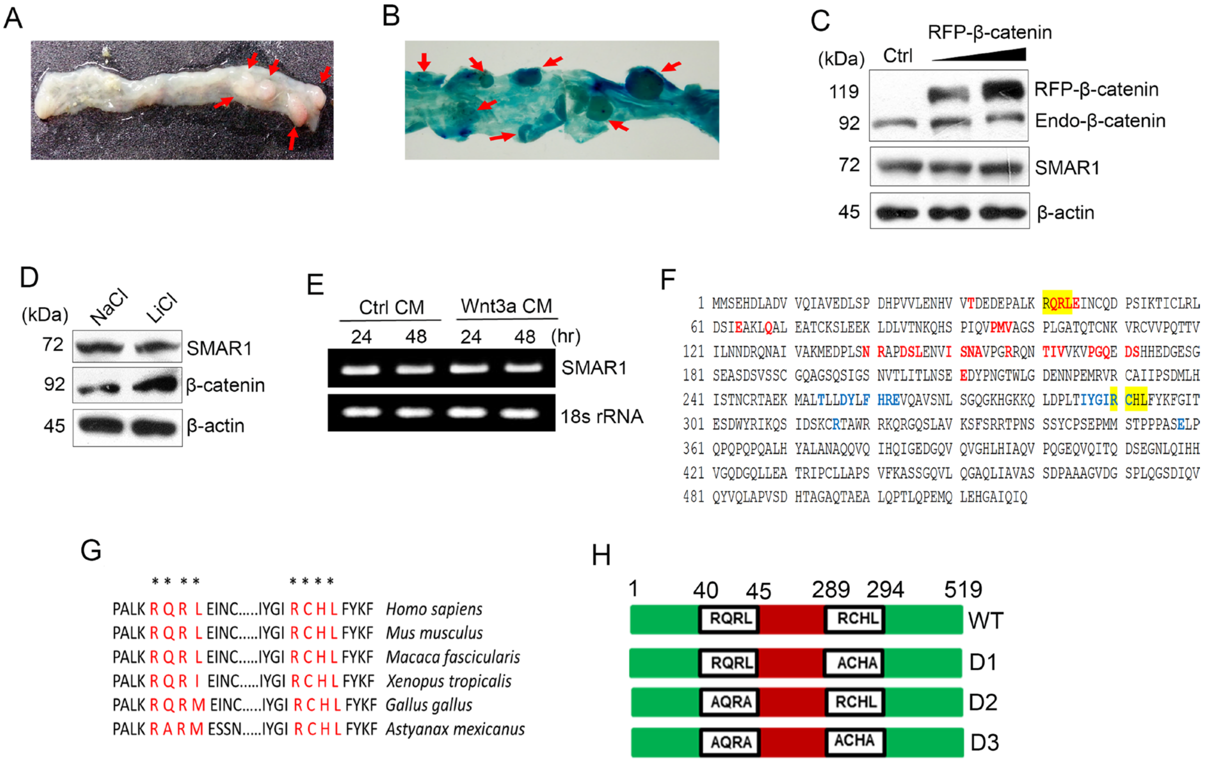

Supplementary Figure 1: SMAR1 is downregulated in Wnt signaling driven CRC. BALB/c mice colon tissue sections showing polyps; (**A**) without staining and (**B**) stained with methylene blue. (**C**) Expression of SMAR1 in RFP-β-catenin overexpressed HCT116 cells. (**D**) SMAR1 expression after treating HCT116 cells with 30 mM NaCl or LiCl for 6 hrs. (**E**) PCR for SMAR1 mRNA after stimulating HCT116 cells with Wnt3a CM. (**F**) Sequence of SMAR1 amino acids obtained from NCBI Reference Sequence: NP_524576.2 showing “RQRL” and “RCHL” highlighted in yellow. (**G**) Sequence of the D-box elements of SMAR1 across various species. (**H**) Representation of the D-box mutations in SMAR1. WT: wild type.

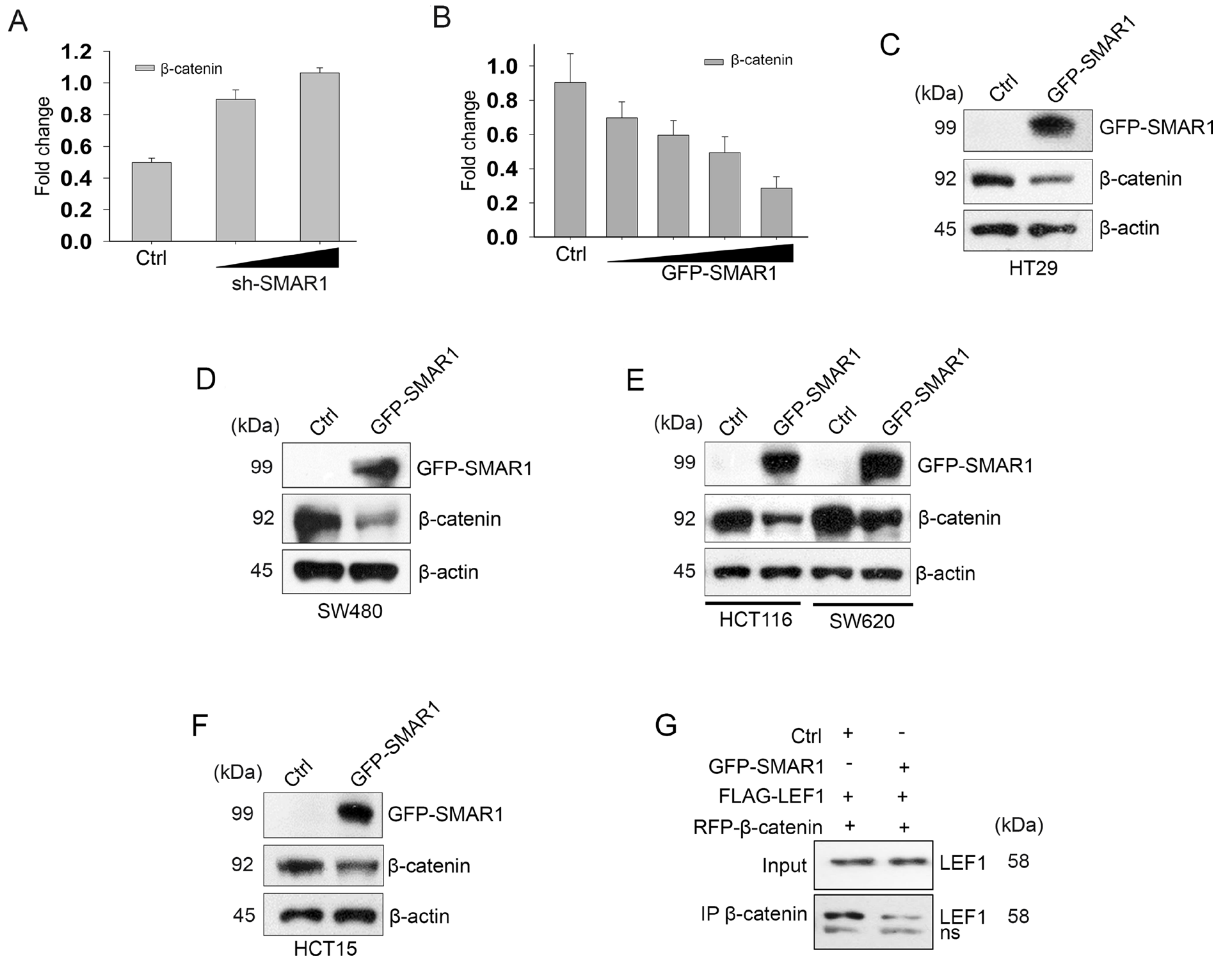

Supplementary Figure 2: SMAR1 inhibits Wnt/β-catenin signaling pathway. Graph representing the densitometry analysis of the blots for β-catenin expression after, (**A**) SMAR1 knockdown and (**B**) GFP-SMAR1 overexpression in HCT116 cells ($n = 3$). β-catenin expression in various CRC cells; (**C**) HT29, (**D**) SW480, (**E**) HCT116 and SW620, and (**F**) HCT15. (**G**) Interaction of LEF1 with β-catenin after overexpressing HCT116 cells with GFP-SMAR1.

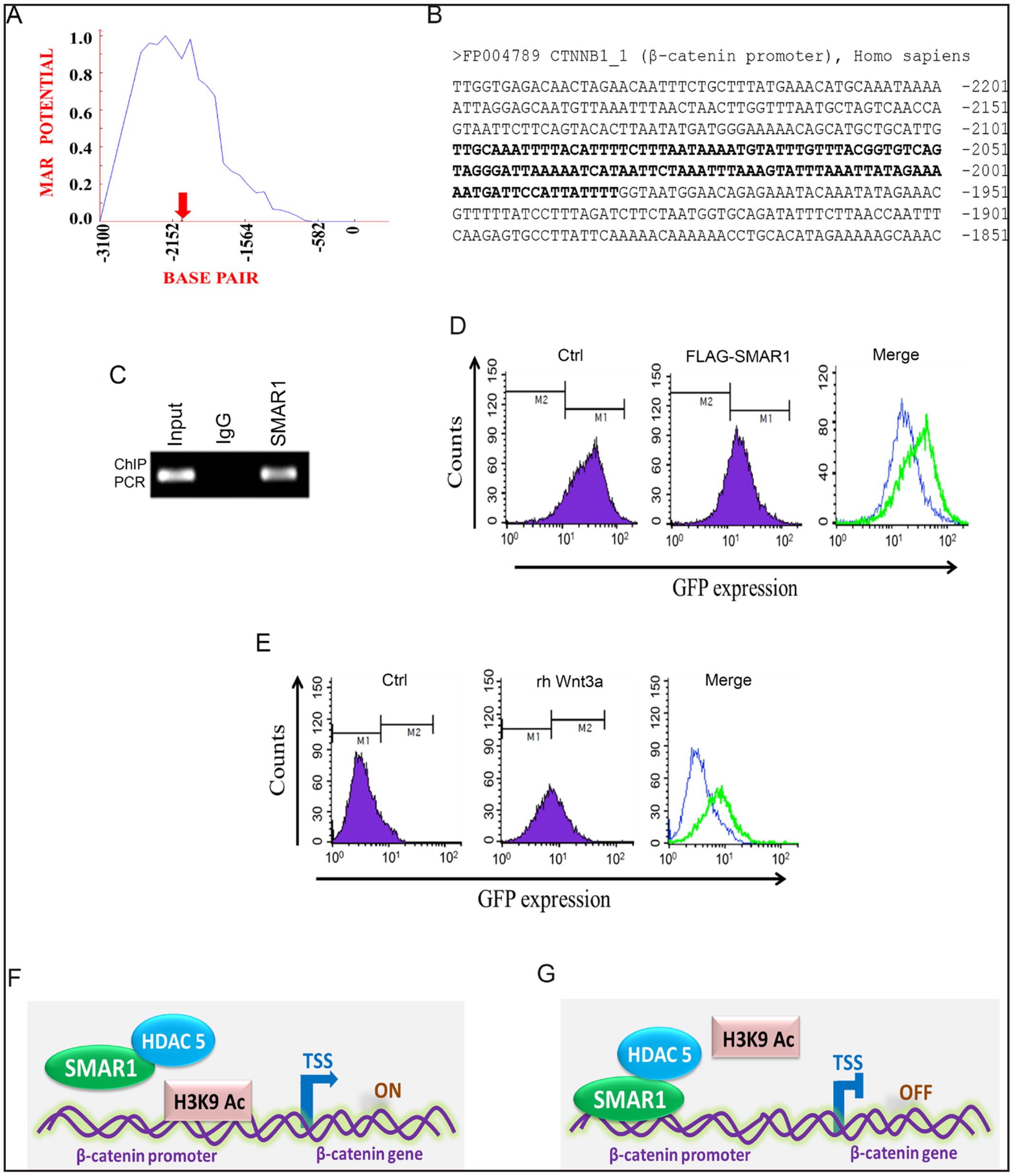

Supplementary Figure 3: SMAR1 suppresses β-catenin promoter activities. (**A**) Graph showing MAR potential on β-catenin promoter generated using MAR-wiz software. (**B**) Sequence of β-catenin promoter showing the putative SMAR1 binding site as predicted by MAR-wiz software. (**C**) ChIP experiment showing SMAR1 occupancy in β-catenin promoter. FACS analysis of pEGFP1-β-catenin promoter expressing GFP in HCT116 cells (mean ± SD, *n* = 3) after; (**D**) Co-transfection with FLAG-vector or FLAG-SMAR1, and (**E**) Treatment with 200 ng/mL rh Wnt3a ligand. Schematic representations of SMAR1, HDAC5 and H3K9 Ac occupancy on β-catenin promoter during: (**F**) SMAR1 knockdown and (**G**) SMAR1 overexpression.

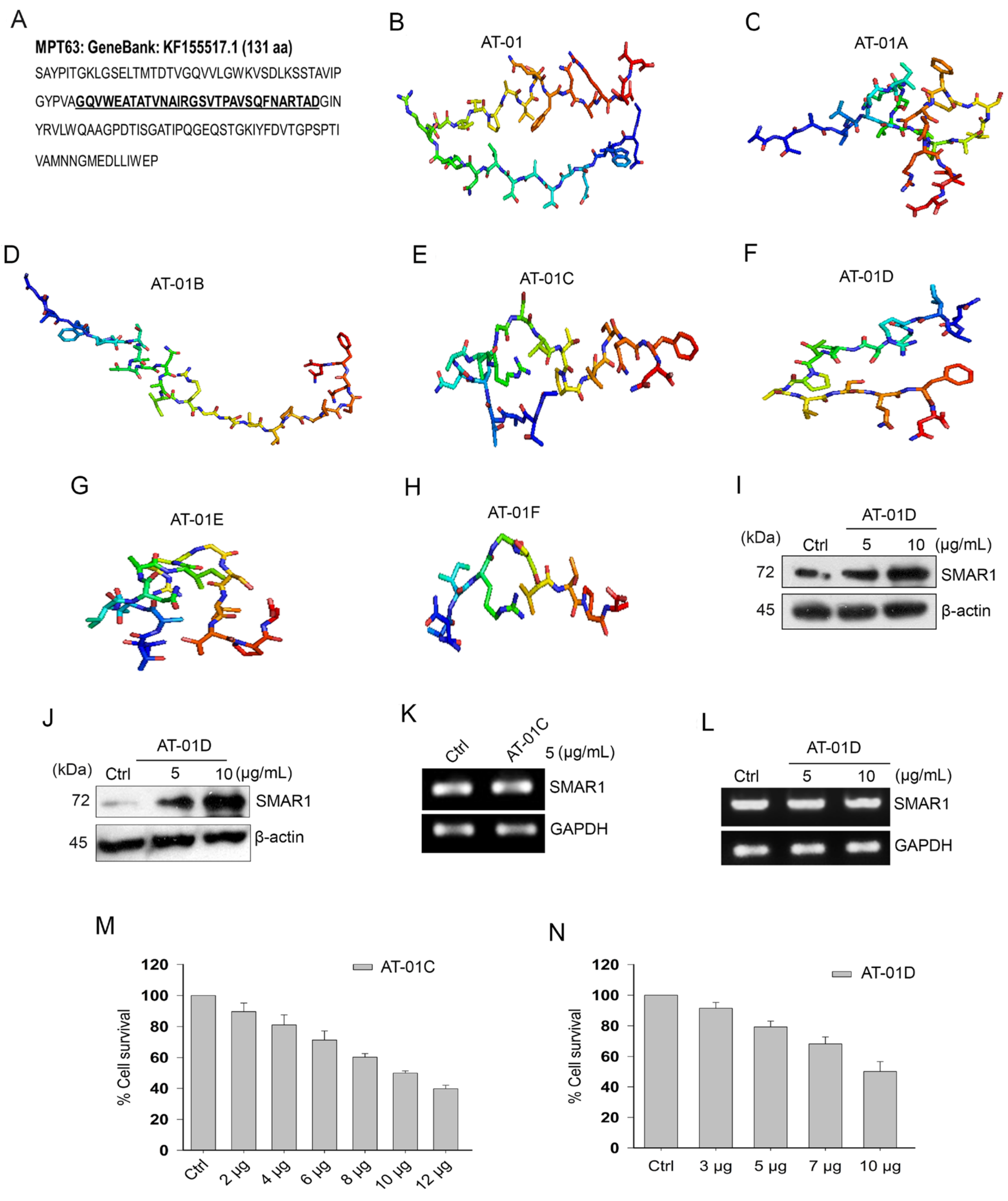

Supplementary Figure 4: Microbial peptides attenuate Wnt/β-catenin signaling. (**A**) MPT63 sequence from which AT-01 peptide is derived. (**B**–**H**) PDB structures of peptides determined using Swiss Model. SMAR1 expression after treating; (**I**) SW480, and (**J**) SW620 cells with AT-01D. PCR showing SMAR1 mRNA levels after treating HCT116 cells with; (**K**) AT-01C, and (**L**) AT-01D. MTT assay (mean ± SD, *n* = 3) in; (**M**) AT-01C, and (**N**) AT-01D treated HCT116 cells for 48 hrs.

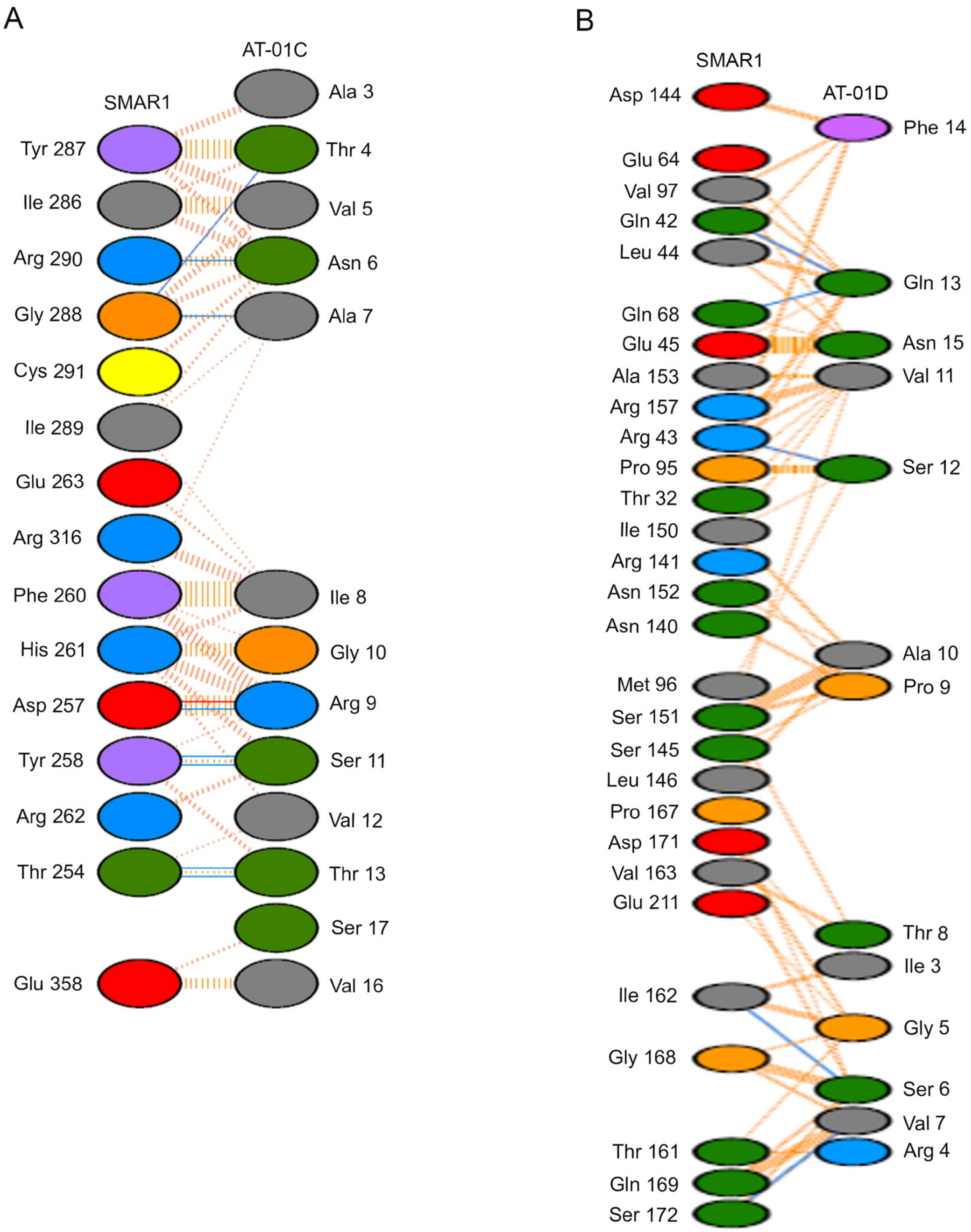

Supplementary Figure 5: AT-01C and AT-01D mask D-box elements of SMAR1. (**A**) Interacting amino acid residues of AT-01C with SMAR1 analyzed using PDBsum. (**B**) Interacting amino acid residues of AT-01D with SMAR1 analyzed using PDBsum. The blue lines indicate hydrogen bonds, red lines indicate salt bridges, and the orange lines indicate non-bonded contacts.
